# Chewing the fat about death with the neighbours

**DOI:** 10.1038/cdd.2016.49

**Published:** 2016-06-13

**Authors:** W B Derry

**Affiliations:** 1 Developmental and Stem Cell Biology Program, The Hospital for Sick Children, Toronto, Ontario, Canada M5G 1X3; 2 Department of Molecular Genetics, University of Toronto, Toronto, Ontario, Canada

Pointed threats, they bluff with scorn

Suicide remarks are torn

From the fool's gold mouthpiece the hollow horn

Plays wasted words, proves to warn

That he not busy being born is busy dying

(Bob Dylan, 'It's Alright, Ma (I'm Only Bleeding)')

There are as many ways to interpret Bob Dylan's lyrics as there are ways that a cell can die. Like Dylan's music, the cell death field has undergone several transformations over the years. In the early days cell death could be divided into three categories based primarily on morphology: Type 1 (programmed cell death or apoptosis), Type 2 (cell death with autophagy), and Type 3 (necrosis).^1, 2^ Genetic approaches in model organisms like *Caenorhabditis elegans* and *Drosophila* uncovered the apoptotic cell death genes and ordered them into pathways,^3, 4^ while biochemical approaches, primarily in cell lines, established the molecular mechanisms by which apoptosis proteins function. Chemical approaches were instrumental in the subsequent identification and study of non-apoptotic forms of cell death, such as necroptosis and ferroptosis.^5, 6^ The two reviews in this issue of *CDD* cover the roads less travelled on the journey to death. They address how signals from neighbouring cells license and fine-tune the apoptotic machinery, and detail the role of lipids in non-apoptotic cell death.

Apoptotic cell death can be activated through either the intrinsic (mitochondrial) or extrinsic (death receptor) pathways, and over the years much effort has been focused on understanding the detailed molecular mechanisms by which the proteins in these pathways function. However, until recently, much less attention has been paid to the influence of neighbouring cells on the death process. It is well known that apoptosis can be regulated by exogenous factors, such as cytokines and nerve growth factor, but there are many differences in how cells die *in vivo* versus *in vitro*. Genetic studies in worms have taught us that the engulfing cell can help promote the execution of a neighbour fated to die, and secreted factors can license the execution machinery.^7, 8, 9, 10^ Work in *Drosophila* has shown that secreted factors collaborate with the core apoptosis pathway to fine-tune its activity, and dying cells (or cells primed for apoptotic death) can stimulate proliferation of their neighbours in a process called compensatory proliferation.^11^ The review by Eroglu and Derry focuses on recent advances in cell non-autonomous mechanisms that regulate apoptosis, a topic that has important implications in development and disease.

While we still have much to learn about apoptosis, a series of papers have recently described alternate ways in which cells can die. These forms of non-apoptotic death, referred to as regulated cell death, involve distinct effector molecules and unique morphological changes. One might even say that we have entered a new renaissance in the cell death field, as we begin to embrace the many forms of cell death that can occur. The review by Matanong, Ko and Dixon tackles the mysterious roles of lipids in regulated cell death. Not only do lipids assist in the formation of pores in cellular membranes that cause their contents to bleed out, but they also serve as signalling molecules in apoptosis and regulated cell death. The plasma membrane is comprised of many different lipid molecules that can be called into action to initiate or assist in various forms of cell death, but we really do not understand how cells utilize these lipids (or 'chew the fat') in these processes. Decoding the logic of how lipids function has important implications in the treatment of numerous diseases that have defects in both apoptotic and regulated cell death (Figure 1).

There are many questions remaining to be answered about non-autonomous apoptosis and the molecular mechanisms of regulated cell death. The intense debate in these areas is reminiscent of the early days of apoptosis research. Autophagy was originally shown to prolong cell survival under nutrient starvation through organelle cannibalism, but more recent studies have shown that it can also trigger the rapid demise of a cell under specific conditions.^12^ This reinforces the old paradigm that life hangs in the balance with death, and the words of Bob Dylan remind us 'that he not busy being born is busy dying'.

## Figures and Tables

**Figure 1:**
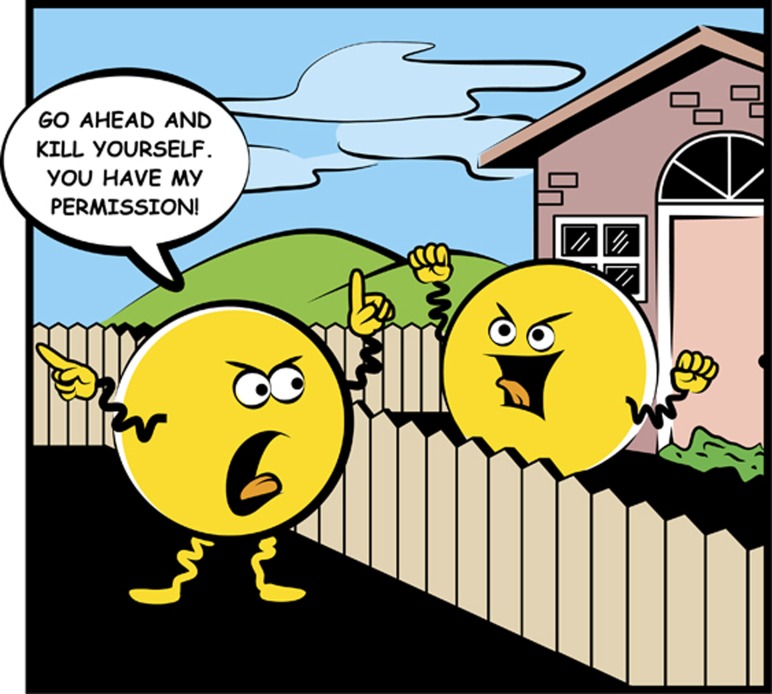
Cells use a variety of molecules, including fatty acids, to assist their neighbours with suicide
